# Optimal Leaf Positions for SPAD Meter Measurement in Rice

**DOI:** 10.3389/fpls.2016.00719

**Published:** 2016-05-26

**Authors:** Zhaofeng Yuan, Qiang Cao, Ke Zhang, Syed Tahir Ata-Ul-Karim, Yongchao Tian, Yan Zhu, Weixing Cao, Xiaojun Liu

**Affiliations:** National Engineering and Technology Center for Information Agriculture, Jiangsu Key Laboratory for Information Agriculture, Jiangsu Collaborative Innovation Center for Modern Crop Production, Nanjing Agricultural UniversityNanjing, China

**Keywords:** chlorophyll meter, rice, measurement method, chlorophyll distribution, nitrogen

## Abstract

The Soil Plant Analysis Development (SPAD) chlorophyll meter is one of the most commonly used diagnostic tools to measure crop nitrogen status. However, the measurement method of the meter could significantly affect the accuracy of the final estimation. Thus, this research was undertaken to develop a new methodology to optimize SPAD meter measurements in rice (*Oryza sativa* L.). A flatbed color scanner was used to map the dynamic chlorophyll distribution and irregular leaf shapes. Calculus algorithm was adopted to estimate the potential positions for SPAD meter measurement along the leaf blade. Data generated by the flatbed color scanner and SPAD meter were analyzed simultaneously. The results suggested that a position 2/3 of the distance from the leaf base to the apex (2/3 position) could represent the chlorophyll content of the entire leaf blade, as indicated by the relatively low variance of measurements at that position. SPAD values based on di-positional leaves and the extracted chlorophyll a and b contents were compared. This comparison showed that the 2/3 position on the lower leaves tended to be more sensitive to changes in chlorophyll content. Finally, the 2/3 position and average SPAD values of the fourth fully expanded leaf from the top were compared with leaf nitrogen concentration. The results showed the 2/3 position on that leaf was most suitable for predicting the nitrogen status of rice. Based on these results, we recommend making SPAD measurements at the 2/3 position on the fourth fully expanded leaf from the top. The coupling of dynamic chlorophyll distribution and irregular leaf shapes information can provide a promising approach for the calibration of SPAD meter measurement, which can further benefit the *in situ* nitrogen management by providing reliable estimation of crops nitrogen nutrition status.

## Introduction

Green leaves are fundamental for the functioning of terrestrial ecosystems (Wright et al., 2004) because green leaf blades are the principal organs of net primary productivity, gas exchange, and evapotranspiration. In practice, the chlorophyll content of leaves is often used to predict the physiological condition of the leaves, as influenced by various natural and anthropogenic factors (Carter, 1994; Zhao et al., 2016).

The methods used for chlorophyll extraction in plants are almost always based on methods that destructively extract leaf tissues using organic solvents (Netto et al., 2005; Yang et al., 2015) such as acetone, ethanol, and dimethyl sulfoxide (DMSO). However, these methods are time consuming and require sophisticated equipment for chemical digestion and other analytical procedures (Wang et al., 2004). Fortunately, the Soil Plant Analysis Development (SPAD, Minolta Camera Co., Osaka, Japan) chlorophyll meter provides a rapid and non-destructive approach that enables users to measure chlorophyll content in the field. This is especially important in monitoring endangered plants (Hawkins et al., 2009) and in determining *in situ* nitrogen (N) status (Arregui et al., 2006; Ziadi et al., 2008; Yuan et al., 2016).

The SPAD meter measures the difference between the transmittance of a red (650 nm) and an infrared (940 nm) light through the leaf, generating a three-digit SPAD value (Uddling et al., 2007). Successful use of the SPAD meter could be affected by many factors, such as cultivar, year, growth stage, leaf thickness, leaf position, and the measurement point on the leaf (Ata-Ul-Karim et al., 2014; Hu et al., 2014), and previous studies have attempted to reduce the influence of these factors. For example, specific leaf weight (SLW) was used to improve the correlation between SPAD values and leaf N concentration (LNC; Peng et al., 1993; Esfahani et al., 2008; Wang et al., 2014). The SPAD Sufficiency Index was calculated to overcome the influence of cultivars, developmental stages, and locations (Varvel et al., 1997; Hussain et al., 2000; Wang et al., 2006). The difference or ratio of di-positional SPAD values was used to eliminate the influence of genotypes and developmental stages (Wang et al., 2006; Lin et al., 2010; Ziadi et al., 2010). In efforts to determine the most representative measurement position, different SPAD measurement methods have been reported. In cereals, depending on the goals of the research, measurements are made at one or more points on either the uppermost fully expanded leaf or the lower leaves. Some studies have attempted to characterize SPAD values at various positions on the leaf blade, including at 1/3, 1/2, 2/3, and 3/4 of the distance from the leaf base to the tip (Turner and Jund, 1994; Matsunaka et al., 1997; Lin et al., 2010; Ziadi et al., 2010), whereas others have characterized SPAD values at more points on the leaf blade (Peng et al., 1993; Lin et al., 2010). However, few systematic analyses have been attempted to optimize the SPAD measurement points on a single leaf blade. Yet results from future studies are totally dependent on the accuracy of these SPAD readings.

SPAD readings are greatly influenced by the specific part of the foliage where the measurements are made, as chlorophyll is not evenly distributed along the leaf blade. In wheat (*Triticum aestivum* L., Debaeke et al., 2006) and corn (*Zea mays* L., Víg et al., 2012), the leaf was divided into several parts for SPAD measurements, and then chlorophyll distribution along the leaf was estimated. This method is appropriate to determine the most suitable position on a leaf blade for chlorophyll measurements, as SPAD values show high variability along the leaf blade from the leaf base to the apex (Chapman and Barreto, 1997). However, for some species with thin leaves, such as rice (*Oryza sativa* L.) and wheat, the special leaf structure might restrict application of the conventional calibration method. In rice, midrib thickness decreases from the leaf base to the tip, while the width of the leaf increases from the base to the midpoint, and then decreases toward the tip (Ata-Ul-Karim et al., 2013). The narrow leaf parts with a thick midrib near the base even cannot cover the entire filed-of-view of SPAD meter, often making the standard deviation of the position 1/3 of the distance from the leaf base higher than that at the midpoint (Lin et al., 2010). These facts considerably limit the usefulness of the conventional calibration method for SPAD measurement adjustments in crops. The flatbed color scanner has the potential to solve this problem.

Research conducted by Eitel et al. (2011) showed that the red and green digital numbers (DN) of the scan image are significantly related to the laboratory-determined total chlorophyll a and b (Chla+b) contents. Thus, spatial chlorophyll distribution across a leaf surface could be mapped using the flatbed color scanner.

Our main objectives were to examine the relationships between DN and SPAD values, to map the chlorophyll distribution of leaves using a flatbed color scanner, and to identify a measurement method capable of detecting the most reliable SPAD values in rice.

## Materials and methods

### Experimental design

Two field experiments were conducted in Jiangsu province of east China from 2013 to 2014, each involving multiple N rates and genotypes (Table 1). Different N application rates (0–375 kg N ha^−1^) in three Japonica rice cultivars, Wuyunjing-19, Yongyou-8, and Wuyunjing-24, and one Indica cultivar, Yliangyou-1, were used to generate various N availability and growth characters. A randomized complete design with three replications was used in the two experiments. The hill spacing was 0.25 × 0.15 m, and two seedlings per hill were transplanted manually in the 5 × 6 m plots. The N fertilizer was applied in the form of urea, with N content of 46%. Urea was applied three times: 50, 20, and 30%, at pre-transplanting, tillering, and booting, respectively.

**Table 1:** **Basic information about the two field experiments**.

**Experiment No**.	**Transplanting date**	**Location**	**Cultivar**	**N rate (kg ha^−1^)**	**Soil type**
Experiment 1 2013	25-June	Zhangjiagang (31°87′N, 120°77′E)	Wuyunjing-19 Japonica Yongyou-8 Japonica	N0(0) N1(90) N2(180) N3(240) N4(360)	Clay loam soil
Experiment 2 2014	15-June	Rugao (32°27′N, 120°76′E)	Wuyunjing-24 Japonica Yliangyou-1 Indica	N0(0) N1(150) N2(225) N3(300) N4(375)	Loam soil

### Plant sampling and N determination

At the tillering (TI), panicle initiation (PI), and heading (HD) stages, five randomly selected hills from each plot were sampled for growth analysis. Fresh leaves were collected and oven-dried at 80°C for 48 h. Leaf dry matter was measured from this material, followed by determination of the LNC using the micro-Kjeldahl method.

### SPAD measurements

SPAD values of the four fully expanded uppermost leaves were determined at the TI, PI, and HD growth stages. The first, second, third, and fourth fully expanded leaves from the top of the plant were designated as LFT1–4, respectively. SPAD readings were taken at three locations: (a) 1/3 of the distance from the leaf base, (b) 1/2 of the distance from the leaf base, and (c) 2/3 of the distance from the leaf base. In the meanwhile, (ab), (ac), (bc) and (abc) mean the combination of the corresponding (a), (b) and (c) positions. Ten randomly selected plants from each plot were measured in the field. For the sake of illustration, Sa, Sb, and Sc represent SPAD readings at leaf locations (a), (b), and (c), respectively. Sabc represents the average SPAD readings of the whole leaf.

### Chlorophyll distribution estimation

#### SPAD values acquisition

To determine the most suitable position on a leaf blade to measure chlorophyll content, two randomly selected hills from plots representing different N application rates (severe N deficiency, 0 kg N ha^−1^; light N deficiency, 150 kg N ha^−1^; normal N supply, 225 kg N ha^−1^) were uprooted. To avoid wilting, plants were stored in plastic buckets containing water. Five representative plants from each bucket were detected in the laboratory using the same operation as in Section SPAD Measurements, and every leaf was marked to facilitate latter analyses.

#### Flatbed color scanner measurements

A flatbed color scanner (Hewlett Packard Scanjet G4050) was used to scan leaf blades after the SPAD measurements, as marked in Section SPAD Values Acquisition. The adaxial sides of the leaves were scanned to obtain eight-bit red, green, and blue digital images with a spatial resolution of 300 DPI (Zhu et al., 2009). Digital images were saved in uncompressed JPEG files. Black and white cardboard (size, 10 × 5 cm) placed at the edge of the scanned area served as a reflectance standard to ensure a constant background for DN values across all scan images (Eitel et al., 2011). Photoshop (Photoshop CS 6, Adobe; San Jose, CA) software was used to identify the red, green, and blue DN values of the scan images.

#### Chla+b determination

After scanning, leaf samples were cut into fine pieces (< 0.25 mm^2^) and chlorophyll was extracted using 96% (v/v) ethanol. Solutions were stored for up to 24 h in darkness (to prevent chlorophyll degradation) until all chlorophyll was extracted, as indicated by white leaf tissue. Chla+b contents were then determined by measuring absorbance at 649- and 665-nm wavelengths on a spectrophotometer (Fritschi and Ray, 2007), and Chla+b concentration (mg/g) was calculated as in Equation (1).

(1) $$Chla+b=\frac{(5.1\times A665+20.04\times A649)\times V}{m}$$

where Chla + b is the chlorophyll a and b concentration (mg/g); A665 and A649 represent the absorbance value at the corresponding wavelength; V refers to the volume (Liter) of chlorophyll solution; m is the weight (Gram) of leaf samples.

### Data analysis

#### Comparison of DN and SPAD values

To examine the relationship between DN and SPAD values, 39 rice leaves were sampled at the TI growth stage. Average DN and SPAD values were computed, and linear regression analysis was performed by least-squares method with IBM SPSS version 21.0.

#### Chlorophyll distribution

The distribution of chlorophyll along the leaf blade was estimated based on DN values to determine the most suitable position on the leaf blade for measuring chlorophyll. In 2014, each scanned blade was divided into 10 equal parts, from the base to the tip, using the slice tool in Photoshop CS 6. Absolute and relative SPAD values derived from the DN values were calculated along the leaf blade under various N rates.

Systematic analyses were performed on representative leaf blades under the N rate of 150 kg N ha^−1^ at the PI growth stage. Calculus algorithm was used to calculate the leaf area of 10 individual parts (Equation 2). The chlorophyll content of each part was estimated according to the corresponding mean SPAD value (Equation 3). Finally, Equation (4) was used to calculate the representative SPAD value (C value) of the whole leaf blade. The representative positions were identified using the distribution equation of the SPAD values by integrating the C value. The conventional method used the arithmetic mean value to represent SPAD values of the leaf. Standard deviations of SPAD values were calculated using SPSS 21.0 and MS-Excel.

(2) f(Ai)=∫f(xi)Δxi  (i=1,2,3…10)

where *f* (Ai) represents the leaf area of a specific leaf part (1–10); *f* (xi) is leaf width based on leaf length (xi) from the leaf base; △xi is the length of a number of equal segments as used in calculus algorithm.

(3) f(SPADi)=SPADi  (i=1,2,3…10)

where *f* (SPADi) is the mean SPAD value of the reference leaf part (1–10); from 1 to 10, the SPAD values of SPADi equal 35.8, 36.5, 39.2, 40.3, 40.6, 39.8, 41.2, 39.4, 37.1, and 34.3, respectively.

(4) $$C=\frac{\sum_{1}^{10}f(Ai)\times f(SPADi)}{\sum_{1}^{10}f(Ai)}\;(i=1,2,3\ldots 10)$$

where C is the representative SPAD value of the whole leaf blade; *f* (Ai) represents the reference leaf area (Equation 2); *f* (SPADi) is the mean SPAD value of the reference leaf part (Equation 3).

#### Chla+b and LNC vs. SPAD readings

The most representative position estimated by the flatbed color scanner was determined based on SPAD measurements. Two N indicators, Chla+b and LNC, were related to the di-positional SPAD values. Their association was estimated by linear and quadratic regression analyses using SPSS21.0.

## Results

### Comparison of DN and SPAD values

**Figure 1:**
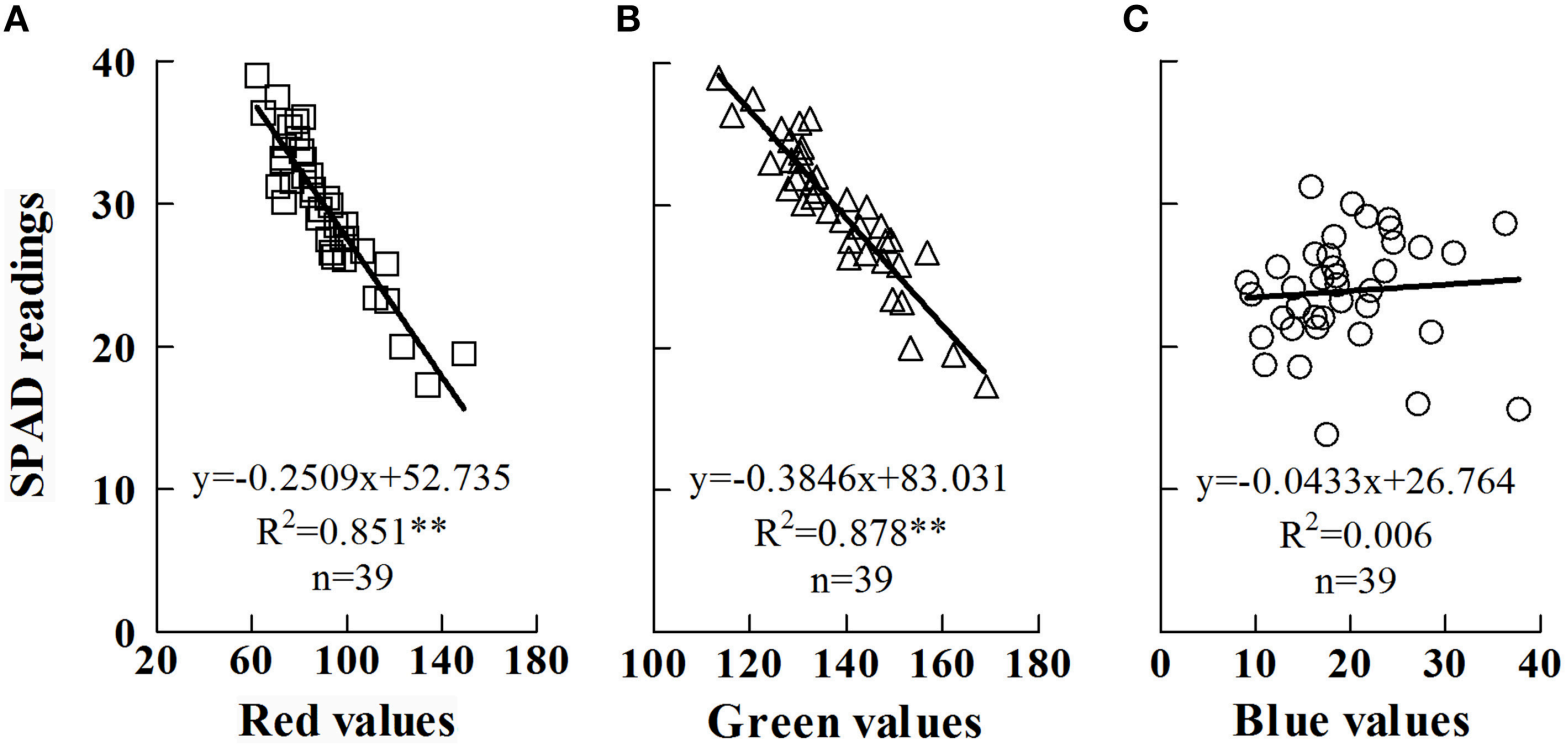
**Relationships between SPAD readings and digital numbers of the red (A), green (B) and blue (C) scanner bands**. Each data point represents a SPAD reading (y-axis value) and digital numbers (x-axis value) of one scanner band at a specific position on the leaf. The specific position was marked after SPAD meter measurement, followed by the scan of a flatbed color scanner (Hewlett Packard Scanjet G4050). And then, the digital numbers were calculated by Photoshop (Photoshop CS 6, Adobe; San Jose, CA). ^**^significant at *p* < 0.01. The analysis was performed by least-squares method with IBM SPSS version 21.0 software.

The DN values of red, green, and blue bands, used individually, were compared to SPAD values. The DN values of those bands had the following relative magnitudes: green>red>blue (Figure 1). Red values ranged from 62.3 to 149.5 (Figure 1A), green values from 113.5 to 169.2 (Figure 1B), and blue values from 9.1 to 37.7 (Figure 1C), under varying N availability. The SPAD values ranged from 17.3 to 39.0, and a linear relationship was fitted between the red, green, and blue values and the SPAD values. The results showed that red and green values accounted for 85.1 and 87.8%, respectively, of the variation in SPAD values, whereas only a small relationship was found between the blue and SPAD values. Since the DN values are ranging from 0 to 256, too low or too high DN values are not appropriate for their wide application. Hence, regression based on red values (Equation 5) was used to estimate the continuous distribution of chlorophyll along the leaf blade in the following section.

(5) $$SPAD=-0.2509^{*}R+52.735$$

where SPAD represents the SPAD value, and *R* is the *DN* value of the red scanner band.

### Mapping the chlorophyll distribution

**Figure 2:**
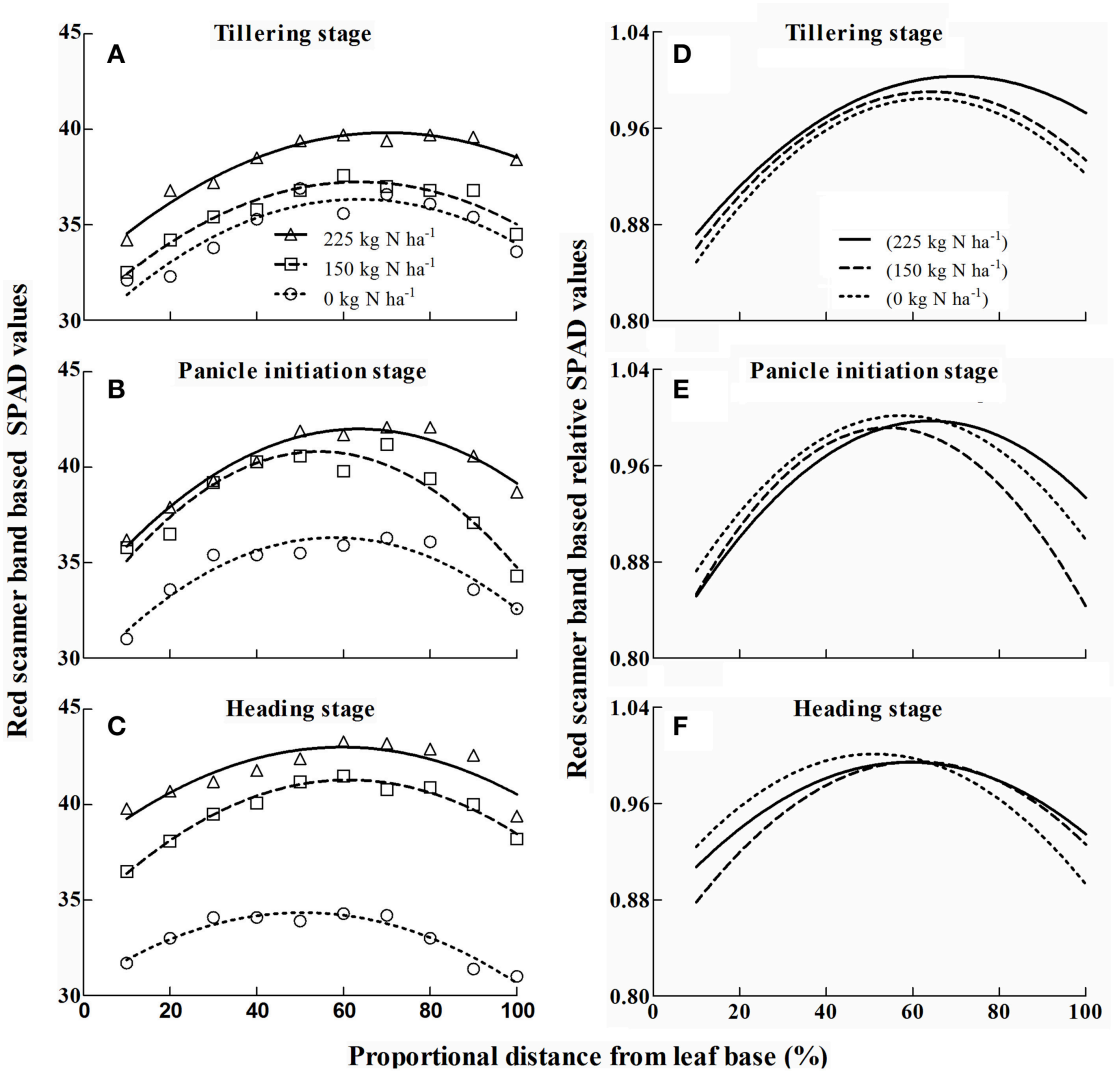
**Variation of red scanner band based absolute (A–C) and relative (D–F) SPAD values along the leaf blade of rice under three N rates (0, 150, 225 kg N ha^−1^) at different growth stages**. Absolute SPAD values **(A–C)** were calculated by Equation (5), in which red scanner band value is the input variable. Normalized SPAD values **(D–F)** were calculated to identify which part of leaf is sensitive to different nitrogen nutrition status. Quadratic regression was fitted between proportional distance from leaf base (%) and red scanner band based SPAD values **(A–C)** and relative SPAD values **(D–F)** by least-squares method with IBM SPSS version 21.0 software.

At the TI, PI, and HD growth stages, the absolute SPAD readings based on the red scanner band (Equation 5) were found to vary with position along the leaf blade under various N rates (Figure 2). A quadratic regression was used to fit the relationship between SPAD values and the proportional distance from the leaf base. At the TI stage (Figure 2A), the differences among N rates were not obvious, whereas they tended to become larger as the plant developed (Figures 2B,C). Although, different SPAD readings were observed at different growth stages under various N rates, the major trends of chlorophyll distribution along the leaf blade were similar.

Generally, SPAD values increased from the base to a position representing 50–60% of the leaf blade, and then they decreased to the leaf apex (Figures 2A–C). To show the trend more clearly, SPAD values were normalized to the largest SPAD value on the leaf blade, and a quadratic regression was used to describe the relationship between the relative SPAD values and the proportional distance from the leaf base. The results showed the ranges of relative SPAD values were 13.39 ± 0.44% (Figure 2D), 13.96 ± 0.74% (Figure 2E), and 10.29 ± 1.64% (Figure 2F) at the TI, PI, and HD growth stages, respectively. In addition, the tip section were more sensitive to the changes of N supply (Figures 2D–F).

**Figure 3:**
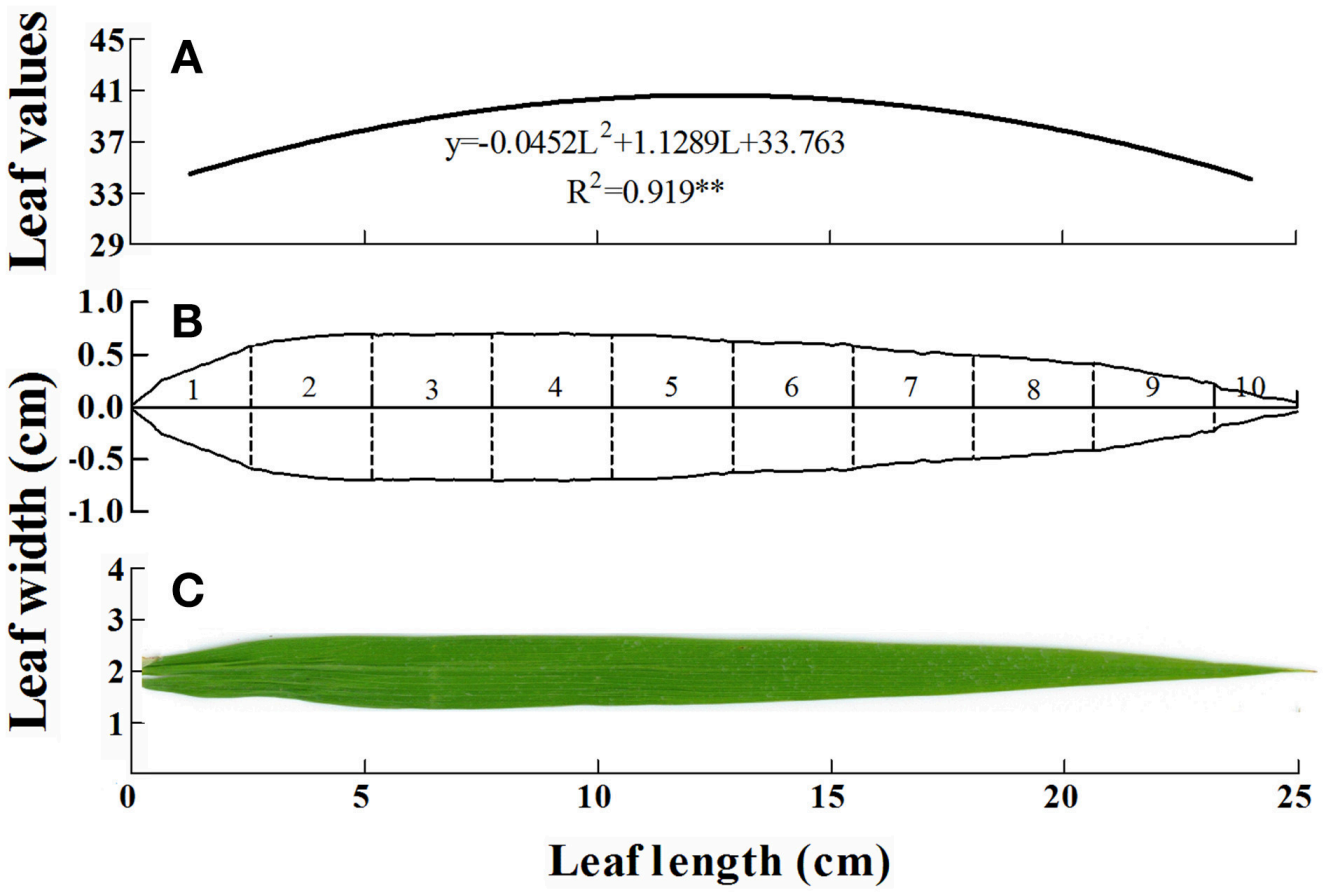
**Leaf characters under light N deficiency (150 kg N ha^−1^) at panicle initiation stage in rice. (A)** The dynamic chlorophyll concentration along the leaf blade can be described by a quadratic regression. ^**^significant at *p* < 0.01. The analysis was performed by least-squares method with IBM SPSS version 21.0 software. **(B)** Leaf shapes were digitalized by Engauge Digitizing software (http://digitizer.Sourceforge.net/). Numbers 1–10 represent ten equal parts of the leaf based on leaf length, which will facilitate the calculation of each part's actual area by calculus method. **(C)** The real leaf blade of rice, from which diagram **(B)** was mapped.

Based on the quadratic equation, the most representative position on the leaf blade should be at two positions, namely near the leaf base and the apex, respectively. To verify this hypothesis, systematic analyses were used to calculate the representative SPAD value of the leaves; this value was 39.11 for the PI growth stage at an N rate of 150 kg N ha^−1^ (Figures 3A–C). Under the same conditions, the representative SPAD value based on the conventional method was 38.42. Both values were entered into the distribution curve of SPAD values (Equation 6). The most representative positions using the present method were at 8.96 and 16.00 cm from the leaf base (about 35 and 63%, respectively), or 1/3 and 2/3 of the distance from the leaf base, respectively.

For the conventional method, the representative positions were 5.21 and 19.76 cm from the leaf base (about 21 and 79%), or ~1/5 and 4/5 of the distance from the leaf base, respectively. The differences between the current and conventional methods indicated that it is essential to conduct systematic analyses of SPAD meter measurements.

(6) $$SPAD=-0.0452L^{2}+1.1289L+33.763$$

where SPAD represents the SPAD value, and L is the distance (cm) from the leaf base (Figure 3A).

**Figure 4:**
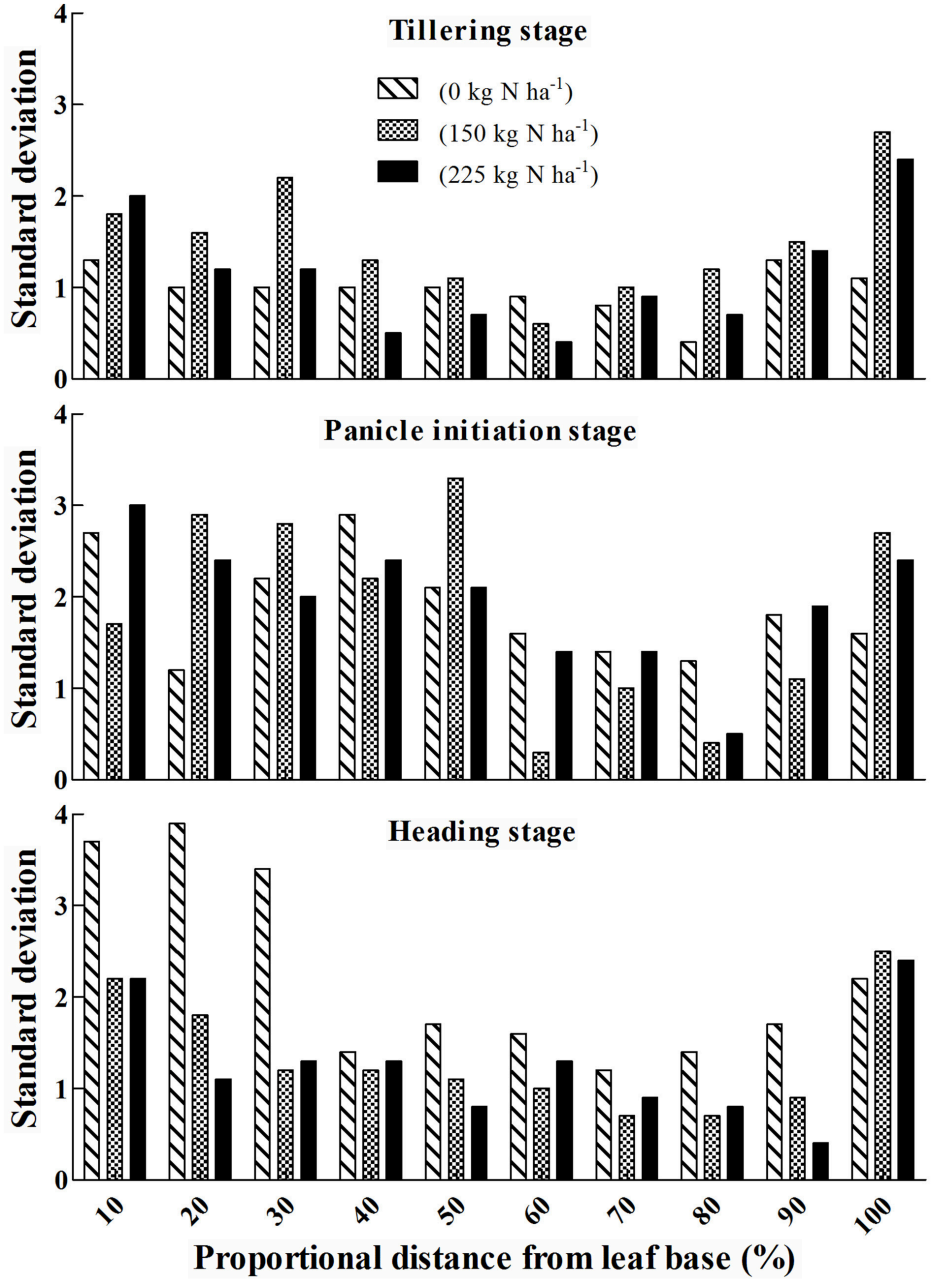
**Variation of SPAD values based on red scanner band along the leaf blade of rice under three N rates (0, 150, 225 kg N ha^−1^) at different growth stages**. Y axis is the standard deviation of absolute SPAD values, which is consistent with Figures 2A–C. Absolute SPAD values were calculated by Equation (5), in which red scanner band value is the input variable.

Taking into account the variation in measurements at different positions, the flatbed color scanner-based measurements were analysed (Figure 4). The standard deviation for different leaf parts decreased from the leaf base to the middle of the leaf, and then increased to the leaf apex (Figure 4). The lowest variance was obtained between 60 and 80% of the distance from the leaf base for all growth stages.

From what has been studied above, we can come to the conclusion that the position 2/3 of the distance from the leaf base (2/3 position) has the following characters: (i) the measurement result on this position is reliable; (ii) this position is more sensitive to N supply; (iii) the variation in measurements at this position is low. Therefore, our results supported 2/3 position as the most suitable position for SPAD measurement in rice.

**Figure 5:**
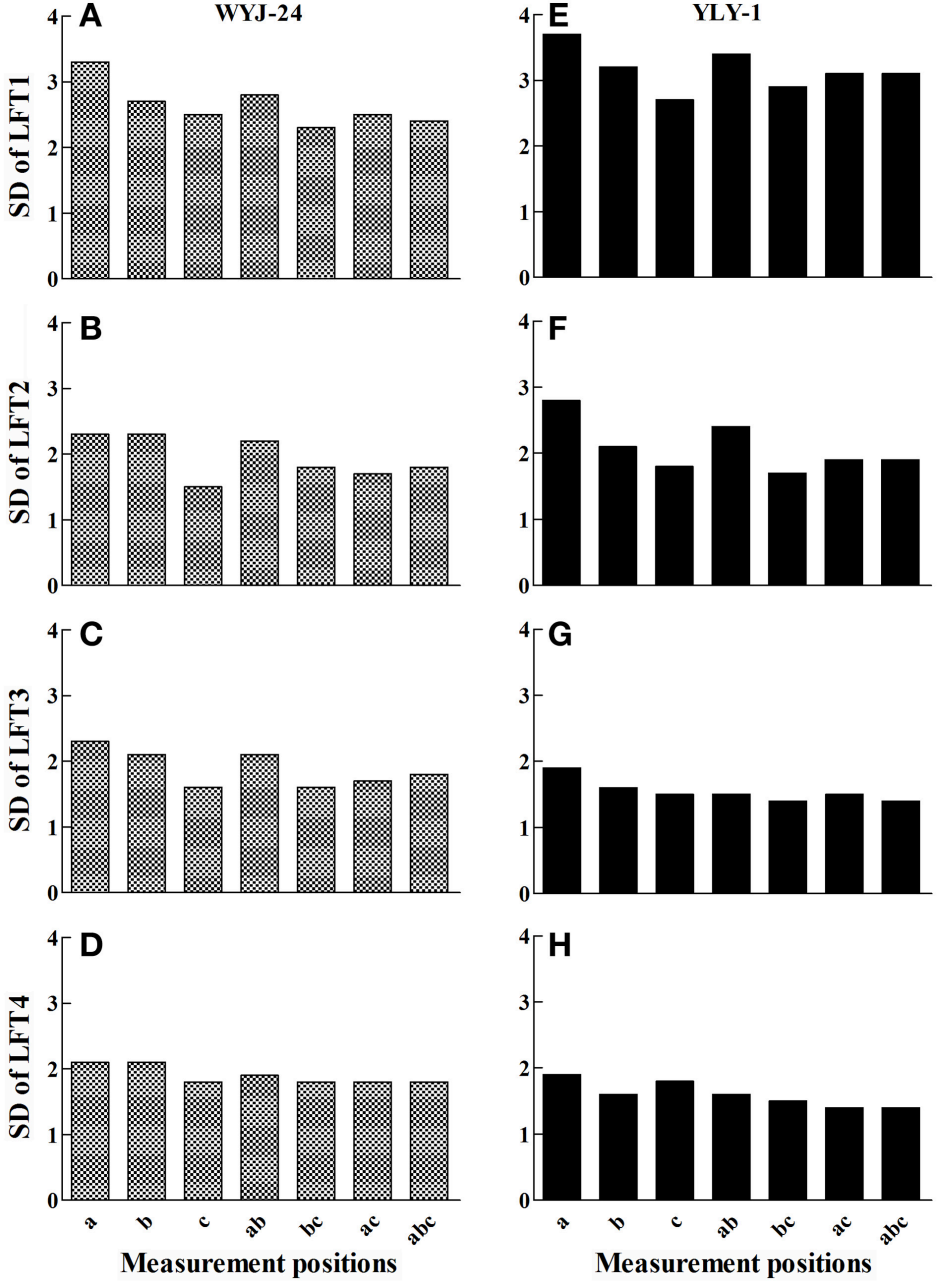
**Variation of SPAD readings at different positions of 20 leaves collected from plants grown under light N deficiency (150 kg N ha^−1^) at panicle initiation growth stage**. WYJ-24, the abbreviation of a Japonica rice hybrid Wuyunjing-24. YLY-1, the abbreviation of an Indica rice hybrid Yliangyou-1. The SD (standard deviation) of the first **(A,E)**, second **(B,F)**, third **(C,G)**, and fourth **(D,H)** fully expanded leaves from top (LFT1-4) were compared at different measurement positions. a–c are leaf positions at the 1/3, 1/2, and 2/3 of the distance from the leaf base, respectively. ab, bc, ac and abc are combinations of the corresponding a–c positions.

A test based on the SPAD meter was performed on di-positional leaves for the N rate of 150 kg N ha^−1^ at the PI growth stage (Figure 5). The standard deviation of the SPAD readings at varying single or combined positions showed that, the variance of the lower leaves tended to be smaller. For single-position measurements, the variance of the c point (2/3 position) on lower leaves was small in both rice cultivars. Thus, this practical test confirmed the previous conclusion that the 2/3 position was the most suitable SPAD measurement position in rice.

### Chla+b and LNC vs. Spad values

**Figure 6:**
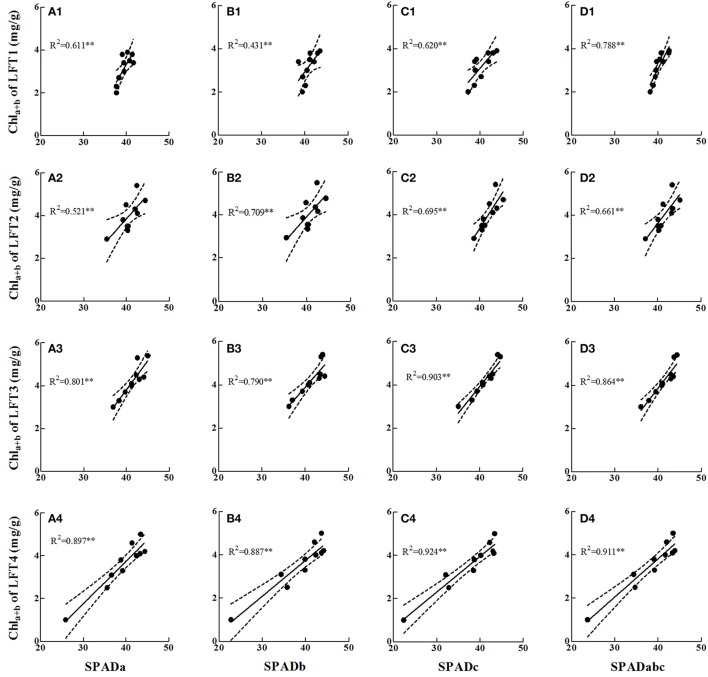
**Relationships between di-positional SPAD readings and chlorophyll concentration (Chla+b) at panicle initiation growth stage**. Di-positional SPAD readings were regressed with chlorophyll concentration of LFT1 **(A1–D1)**, LFT2 **(A2–D2)**, LFT3 **(A3–D3)**, and LFT4 **(A4–D4)**. LFT1-4 represent the first, second, third, and fourth fully expanded leaf from top. SPADa, SPADb and SPADc are the SPAD readings detected at the 1/3, 1/2, and 2/3 of the distance from the leaf base, respectively. SAPDabc is the average values of SPADa, SPADb and SPADc. ^**^significant at *p* < 0.01. The solid line denotes the linear regression and the dotted lines represent the confidence bands (*p* = 0.95). The analysis was performed by least-squares method with IBM SPSS version 21.0 software.

A close relationship was found between SPAD values and the extracted Chla+b content (Figure 6), with coefficients of determination (*R*^2^) ranging from 0.431 to 0.924 (*p* < 0.01). Chla+b ranged from 1.0 to 5.4 mg g^−1^, and the SPAD values ranged from 22.8 to 45.6. The LFT2 and 3 leaves (Figures 6A2/A3,B2/B3,C2/C3,D2/D3) tended to contain more Chla+b than the higher (LFT1, Figures 6A1–D1) and lower (LFT4, Figures 6A4–D4). The *R*^2^ value increased from LFT1 to LFT4. As expected, the most suitable SPAD measurement position was observed at the c point (i.e., approximately the 2/3 position).

As the lower leaves tended to be more sensitive to the N status of rice, we examined the correlation between the average SPAD values of LFT4 (SPAD4) and the SPAD values at the c point (SPAD4#) with LNC. Quadratic regression analysis was used to describe this association (Table 2). Statistically significant relationships (*p* < 0.01) were observed in different cultivars at both the PI and the HD growth stages. However, SPAD4# could efficiently improve this correlation for all rice cultivars, except for WYJ-19 at the PI growth stage. These results demonstrated that the most suitable SPAD measurement position was the c point. Therefore, the c point (approximately the 2/3 position) on LFT4 could be used as the most suitable position for SPAD meter measurements in rice.

**Table 2:** **Quadratic regression analyses between SPAD indicators and leaf nitrogen concentration (LNC) at different growth stages**.

**Growth stage**	**Cultivar**	**X**	**Data (*n*)**	**Regression equation**	**R^2^**
Panicle initiation	WYJ-24	SPAD4	15	LNC = 0.0003X^2^ + 0.051X + 0.199	0.729^**^
		SPAD4#	15	LNC = 0.004^2^ − 0.2257X^2^+ 5.4525	0.743^**^
	YLY-1	SPAD4	15	LNC = 0.003X^2^ + 0.0161X + 1.538	0.621^**^
		SPAD4#	15	LNC = 0.0016X^2^ −0.0668X + 2.7981	0.782^**^
	WYJ-19	SPAD4	15	LNC = 0.016X^2^ −1.146X + 22.79	0.653^**^
		SPAD4#	15	LNC = 0.0117X^2^ − 0.8031X + 15.909	0.587^**^
	YY-8	SPAD4	15	LNC = 0.0051X^2^ − 0.3109X + 6.5303	0.721^**^
		SPAD4#	15	LNC = 0.0085X^2^ − 0.5734X + 11.55	0.871^**^
Heading	WYJ-24	SPAD4	15	LNC = 0.0087X^2^ − 0.6617X + 14.901	0.732^**^
		SPAD4#	15	LNC = 0.0098X^2^ − 0.7388X + 15.998	0.772^**^
	YLY-1	SPAD4	15	LNC = 0.0062X^2^ − 0.411X + 8.756	0.813^**^
		SPAD4#	15	LNC = 0.0102X^2^ − 0.722X + 14.939	0.865^**^
	WYJ-19	SPAD4	15	LNC = −0.0066X^2^ + 0.6322X − 12.493	0.759^**^
		SPAD4#	15	LNC = −0.0037X^2^ + 0.3919X − 7.3598	0.879^**^
	YY-8	SPAD4	15	LNC = 0.0029X^2^ − 0.0895X + 1.1076	0.594^**^
		SPAD4#	15	LNC = 0.0204X^2^ + 1.4466X + 27.33	0.806^**^

** *F-test statistical significance at the 0.01 probability level*.

## Discussion

The SPAD meter is a promising tool for diagnosing the N status of crops (Lemaire et al., 2008). A large number of studies have reported a good correlation between SPAD indicators and the N nutrition index (Prost and Jeuffroy, 2007), plant N concentration (Giletto and Echeverría, 2013), LNC (Errecart et al., 2012), and extracted chlorophyll content (Uddling et al., 2007). However, SPAD readings are affected by many factors. Numerous attempts have been made to improve the reliability of SPAD meter readings based N diagnosis in crop production (Peng et al., 1993; Debaeke et al., 2006), but the quality of the original measurements was not considered. Unfortunately, the final analysis was totally dependent on the original measurements. Therefore, it is necessary to conduct systematic analyses before applying the SPAD meter to use it more efficiently.

In this study, we used a flatbed color scanner to indirectly estimate chlorophyll distribution along the leaf. The DN values of the red and green bands, used individually, were significantly correlated with SPAD readings on the same leaf, which is consistent with previous reports (Eitel et al., 2011). Previous research usually used only the SPAD meter to estimate chlorophyll distribution along the leaf, and then recommended a suitable position for subsequent SPAD meter measurements (Víg et al., 2012). This method is efficient for species with wide leaves, such as corn and potato (*Solanum tuberosum* L.), but SPAD measurements might be useless for species with thin leaves, such as wheat and rice. This is because the sampling area of the SPAD meter is limited on thin leaves (Lin et al., 2010), as the narrow parts even cannot cover the entire sensor field of view of the SPAD meter. Under such situations, a flatbed color scanner could provide an alternative way to accurately estimate chlorophyll distribution along the leaf. The flatbed color scanner is more reliable than the SPAD meter in mapping chlorophyll distribution for two reasons: (i) the samplings can be continuous, with precise interstices, and (ii) the samplings can accurately avoid the leaf veins and margins.

This study's use of quadratic regression of SPAD values on the proportional distance from the leaf base was similar to the descriptions by Chapman and Barreto (1997) in corn and by Debaeke et al. (2006) in wheat. This might suggest that in different plant species, chlorophyll distribution along the mature green leaf is similar. Based on the characteristics of the quadratic equation, the most representative position on the leaf should be located between the basal, apex, and central parts. Our systematic analyses showed that the two potential positions to represent whole leaf chlorophyll status were positions at about 35 and 63% of the distance from the leaf base, roughly equal to the 1/3 and 2/3 positions. These results were different from those derived from the conventional method (about 20 and 80% of the distance from the leaf base), based on the same dataset. This is because the conventional method did not consider the proportions of different leaf parts. According to Víg et al. (2012), two qualities are required for the most suitable measurement point on a single leaf blade: (i) the chlorophyll of the specific area is representative, and (ii) the measurement variance in the specific area must be low. Thus, taking into consideration both requirements (Figures 4, 5), the most suitable and representative position for SPAD meter measurements on the leaf blade of rice is at the 2/3 position.

In our study, the analysis systematically considered the dynamic chlorophyll distribution, irregular shape of the leaf, and measurement variance, all of which were improvements over the conventional method (Víg et al., 2012). This is because the conventional method considers only the measurement variance (Debaeke et al., 2006; Lin et al., 2010) or the mean SPAD values of multi-point measurements (Víg et al., 2012). Moreover, the latter value is just the arithmetic mean of the measurement results, whereas the shape of the crop leaf is usually irregular. Therefore, the method provided from this study is a promising way to identify a suitable position for SPAD meter measurements and to improve the quality of the original SPAD readings for subsequent analyses.

The SPAD meter is a leaf-clip apparatus. Di-positional leaves are compared to predict the N status of the plant (Turner and Jund, 1994; Wang et al., 2006; Lin et al., 2010). The same research at the PI growth stage (Figure 6) showed that the lower leaves tended to be more sensitive to the N status of a plant. There are two potential reasons for this phenomenon (i) sufficient time is still needed for stabilization of leaf color after complete unfolding of the uppermost leaf (Matsunaka et al., 1997), and (ii) upper leaves tend to use remobilized N from a lower leaf and stems for growth during periods of N shortage (Zhou and Wang, 2003). In addition, measurement positions including single and averaged multiple positions were also examined in the field (Figure 6). The results showed that the c point (about 2/3 of the distance from the leaf base; Figures 6C1/C2/C3/C4) in the four uppermost fully expanded leaves was the most stable and representative position. Because the 2/3 position on lower leaves was more sensitive to the N status of a plant, the 2/3 position and average SPAD values of LFT4 (SPAD4# and SPAD4, respectively) were examined by LNC (Table 2). As expected, SPAD4# provided a better prediction of leaf N status in rice.

## Conclusion

In our study, a flatbed color scanner was used to map the shape and dynamic distribution of chlorophyll in mature rice leaves. Two potential positions (1/3 and 2/3 positions) were calculated using the calculus method, which takes into account the dynamic distribution of chlorophyll along the leaf, as well as the irregular leaf shape.

Measurement variance studies showed that the 2/3 position had the lowest variance at different growth stages. Also, *in situ* examinations indicated that the SPAD values at the 2/3 position on LFT4 were more reliable in predicting the N status of the plant than were those on other leaves. Therefore, we recommend the 2/3 position on LFT4 as the most suitable measurement position in rice. Although, we hope the methodology established in this paper can promote the most suitable measurement analysis of the SPAD meter in different crops, further research is still needed to verify and improve this methodology.

## Author contributions

ZY performed experiments with support by KZ and SA; XL, QC, YT, YZ and WC provided advice and edited the manuscript; XL and ZY planned experiments and ZY wrote the manuscript. All authors read and approved the final manuscript.

## Funding

The work was supported by the National High-Tech Research and Development Program of China (2013AA100404), Special Program for Agriculture Science and Technology from the Ministry of Agriculture in China (201303109), the Priority Academic Program Development of Jiangsu Higher Education Institutions of China (PAPD), Natural Science Foundation of Jiangsu Province (BK20150663), and the Three-new Agriculture Project of Jiangsu Province (SXGC[2014]304).

### Conflict of interest statement

The authors declare that the research was conducted in the absence of any commercial or financial relationships that could be construed as a potential conflict of interest.

## Abbreviations

Chla+b: chlorophyll a and b contents (mg g^−1^)
DN: digital number
HD: heading
LFT4: fourth fully expanded leaf from top
LNC: leaf nitrogen concentration
N: nitrogen
PI: panicle initiation
TI: tillering.
